# 

**Published:** 1876-04

**Authors:** 


VOL. XXXIII.
NUMBER 4.

April, 1876.

NEW SERIES.
VOL. I.
NUMBER 4.

### THE CHICAGO MEDICAL Journal and Examiner

PUBLISHED MONTHLY.

**EDITOR:**
WILLIAM H. BYFORD, A.M., M.D.

**ASSOCIATE EDITORS:**

JAMES H. ETHERIDGE, M.D. | JAS. NEVINS HYDE, A.M., M.D.

NORMAN BRIDGE, M.D. | FERD. C. HOTZ, M.D.

Published under the auspices of the
*CHICAGO MEDICAL PRESS ASSOCIATION.*

**TERMS—FOUR DOLLARS PER ANNUM, IN ADVANCE.**
**POSTAGE FREE.**

CHICAGO:
W. B. KEEN, COOKE & CO., Publishers,
NOS. 113 AND 115 STATE STREET.

